# Determining the migration duration of rice leaf folder (*Cnaphalocrocis medinalis* (Guenée)) moths using a trajectory analytical approach

**DOI:** 10.1038/srep39853

**Published:** 2017-01-04

**Authors:** Feng-Ying Wang, Fan Yang, Ming-Hong Lu, Shan-Yu Luo, Bao-Ping Zhai, Ka-Sing Lim, Caitríona E. McInerney, Gao Hu

**Affiliations:** 1 College of Plant Protection, Nanjing Agricultural University, Nanjing, China; 2 Plant Protection Research Institute, Guangxi Academy of Agricultural Science and Technology, Nanning, China; 3 Vegetable Research Institute, Wuhan Academy of Agricultural Science and Technology, Wuhan, China; 4 Division of Pest Forecasting, China National Agro-Tech Extension and Service Center, Beijing, China; 5 Agro-Ecology Department, Rothamsted Research, Harpenden, Hertfordshire, UK; 6 Computational and Systems Biology, Rothamsted Research, Harpenden, Hertfordshire, UK

## Abstract

Many moths finish their long distance migration after consecutive nights, but little is known about migration duration and distance. This information is key to predicting migration pathways and understanding their evolution. Tethered flight experiments have shown that ovarian development of rice leaf folder (*Cnaphalocrocis medinalis* [Guenée]) moths was accelerated and synchronized by flight in the first three nights, whereby most females were then matured for mating and reproduction. Thus, it was supposed that this moth might fly three nights to complete its migration. To test this hypothesis, 9 year’s field data for *C. medinalis* was collected from Nanning, Guangxi Autonomous Region in China. Forward trajectories indicated that most moths arrived at suitable breeding areas after three nights’ flight. Thus, for *C. medinalis* this migration duration and distance was a reasonable adaptation to the geographic distribution of suitable habitat. The development of female moth ovaries after three consecutive night flights appears to be a well-balanced survival strategy for this species to strike between migration and reproduction benefits. Hence, an optimum solution of migration-reproduction trade-offs in energy allocation evolved in response to the natural selection on migration route and physiological traits.

Many insects undertake long-distance migrations to avoid adverse conditions and exploit temporary or patchy habitats^1^,^2^,^3^. Due to their limited flight capabilities, they perform their annual migration journey with the help of the wind at heights of a hundred or even a thousand meters^1^,^2^,^4^,^5^. Their flight heights are much too high to be seen by the naked eye. Besides, many of them fly in the dark^1^,^2^,^6^,^7^. Most insect windborne migrations are therefore invisible to a ground based observer. Although the entomological radar is a very useful tool to observe the movement of insects at high altitude, the farthest distance covered by the radar is only ten kilometers at horizon distance and therefore much less than the migratory insect’s flight distance^6^. Recent technological advancement of high-resolution Global Positioning Systems and other sensors such as miniaturized tracking tags have dramatically improved our ability to describe animal movements. To date, only a few large day-flying insects have been tracked at low heights at distances of ten kilometers ranges^8^,^9^,^10^,^11^. Very little is therefore known about the distance covered by insects during their migration. How far an insect can migrate is a fundamental question that needs to be answered to predict insect migration pathways.

Many small insects perform non-stop migration and complete their long-distance journey as one continuous flight^1^. For example, the flight duration of the brown planthopper was estimated to be over 36 h or more^12^,^13^. Based on previous studies, many nocturnal moths always take off at dusk, stop flight at the next dawn, and then take-off again at the next dusk. They refuel themselves by feeding on nectar to build up the energy needed for their next flight. They fly on consecutive nights in order to try to complete their long distance migration in as short a period as possible. This type of migration is known as multi-stop migration, and the Armyworm (*Mythimna seperata* [Walker]), Silver Y moth (*Autographa gamma*) and the rice leaf roller (*Cnaphalocrocis medinalis* [Guenée]) are known to adopt this migration strategy^5^,^14^,^15^. Studies have shown that these nocturnal migrants are capable of travelling up to 300 km per night^4^,^5^,^7^, thus any inaccuracy in predicting the number of flight nights will produce a dramatic error in predicting the migration pathway. Tethered flight experiments have been carried out to test the flight capability of migratory moths. It has been shown that *C. medinalis* can fly 4–5 and even up to 9 consecutive nights^16^. Results probably do not reflect the flying behavior of moths in the wild because tethered moths were tested in an unnatural setting^17^,^18^.

For a small migratory insect, investments in flight organs are costly and it is widely accepted that this is a costly strategy in terms of reproduction output^19^. Competition for limited internal resources lead to physiological management of migration-reproduction trade-offs in energy allocation^20^,^21^. Johnson (1963, 1969) described this migration-mediated reproductive cost as the ‘oogenesis–flight syndrome’. Previous observations have shown that migration in many insects is restricted to the post-teneral, pre-reproductive period^22^,^23^,^24^. Therefore, approximate flight capacity might be ascertained from studies on the oogenesis-flight relationship. A case in hand is *C. medinalis*, a major migratory insect pest of rice that has had serious outbreaks in many Asian countries in recent decades, especially in China^14^,^15^,^25^. Their migration/reproduction trade-off and the oogenesis-flight relationship has been well studied^25^,^26^. The ovarian development of young female *C. medinalis* moths was accelerated and oviposition was synchronized by flight in the first three nights^25^ (F. Yang & G. Hu, unpublished). Ovaries of most females was matured for mating and reproduction after three nights’ flight^25^ (F. Yang & G. Hu, unpublished). Thus, this may indicate that this moth flies three nights to complete its migration. In addition, no significant differences in mating frequency and lifetime fecundity were observed among the unflown controls and those that had flown for 1–3 nights, but moths which took flight more than 3 consecutive nights had lower mating frequency and lifetime fecundity^25^. In other words, there were no negative influences of flight on reproduction if female moths flew 3 or fewer nights.

As the ‘oogenesis–flight syndrome is produced from responses to natural selection in energy allocation^27^, flight duration can be considered the optimum solution for migrants to complete their migration and maximize the benefits for their reproduction. Therefore, flight duration can be used to estimate migration distance. Flight duration is also directly affected by the distribution of suitable habitat in both space and time. Most migratory moths should be able to reach their preferred habitat within their flight capability time. Thus, three consecutive nights’ flight should be a reasonable amount of time for *C. medinalis* to complete its migration and arrive at suitable habitat. To test this hypothesis, 9-years field data were collected for *C. medinalis* from Nanning City, Guangxi Autonomous Region (1978, 1979, 1980, 1981, 2004, 2005, 2007, 2010 and 2011). The probabilities for *C. medinalis* to successfully arrive in its expected destination were calculated by forward trajectories (Fig. 1). By exploring the relationship between migration, reproduction and the geographic distribution of its habitat, in this study we aim to gain a better understanding of insect migration and its consequences.

## Results

Population abundance data from field surveys indicated that there was an emerged peak of *C. medinalis* adult moths from the end of May to the middle June in Nanning each year (Fig. 2). During this peak, rice plants were at flowering stage and were not suitable for *C. medinalis* larva to feed on (Table 1). Females with ovarian development Level I - II accounted for more than 65% of the female population in every year (Table 2). This suggested that most adults emerged from the local population and would emigrate before their ovaries matured, according to the criteria described by Zhang *et al*.^26^.

**Figure 1:**
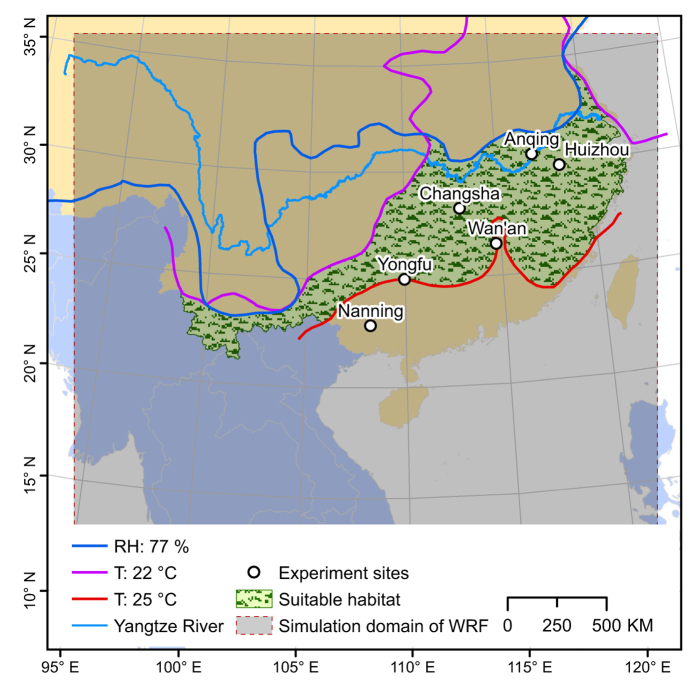
Study area examined using the WRF model to identify suitable habitat for migratory *C. medinalis* and locations of experimental sites in China. Suitable habitat was determined by examining temperature, relative humidity and rice plant data considering that (i) the optimal temperature and relative humidity range for *C. medinalis* eggs is 22–31 °C and 77–100%, respectively^48^ and (ii) only young rice plants are suitable. Early season rice in southern China is most sensitive to low temperatures during the heading stage^52^ and rice plants should be too old for *C. medinalis* if they grew in temperatures greater than 25 °C. Suitable habitat for migratory *C. medinalis* is to areas where the average temperature is 22–25 °C and the relative humidity is greater than 77%, as indicated on the map. (The map was produced using ArcGIS 10.2 software, http://www.arcgis.com/features/).

**Figure 2:**
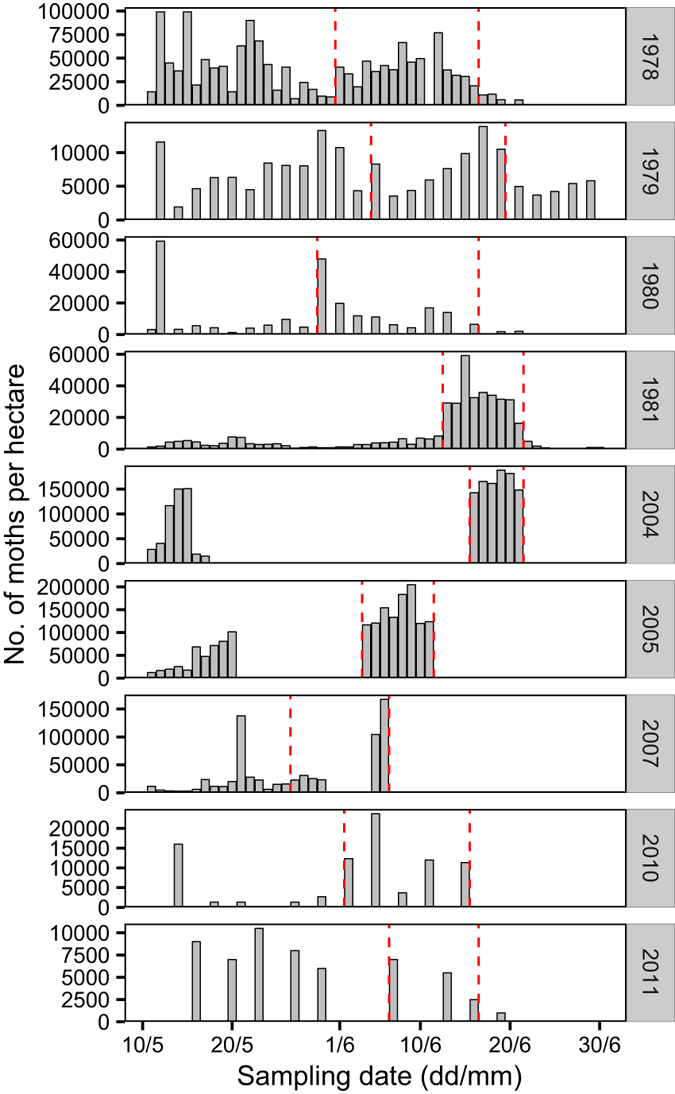
Population dynamics of *C. medinalis* moths in Nanning, China. Results of the field survey performed daily in 1978, 1981, 2004, 2005 and 2007, every two days in 1979, 1980, and every 3 days in 2010 and 2011. In 2007, the field survey was performed from 11 May to 6 June but the data from 31 May to 4 June is missing. The period between the 2 dashed red lines indicates the emigration period.

Distributions of temperature and relative humidity in early and middle June indicated that the expected destination of migratory *C. medinalis* was located in the lower and middle reaches of the Yangtze River (Fig. 1). The rice planting schedules surveyed at Yongfu, Changsha, Huizhou and Huaining indicated that farmers practice both single- and double-cropping in this region (see Table 1). The early season rice in this area was at booting stage during this period and was harvested from middle July to early August. The late season rice was transplanted after the harvest of early rice. Single cropping rice planting technique is also known as middle season rice planting. Middle season rice is generally planted later than early rice. The plants of middle season rice were transplanted from late May to early July irregularly and were at different stages from seedling to tillering in early and middle June (Table 1). In summary, the progeny produced from migratory *C. medinalis* would be able to find suitable host rice plants at the correct growth stage located in the lower and middle reaches of the Yangtze River during early and middle June.

**Figure 3:**
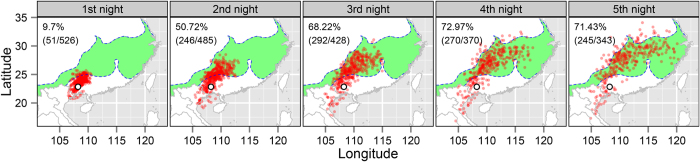
Distributions of endpoints of forward trajectories starting from Nanning, China. Red dots indicate the endpoints of the forward trajectories, and the green-filled region delineates the potential suitable habitat of *C. medinalis* (Fig. 1). The percentage of endpoints that successfully landed in suitable habitat is given with the number of all valid endpoints shown in parenthesis. Trajectory analysis was determined over 5 consecutive nights. (The map is made by R software, https://www.r-project.org/).

Forward trajectories were calculated for 110 events and the distributions of endpoints are shown in Fig. 3. Most trajectories arrived after three or four consecutive nights to within the area delineated as suitable habitat located in the lower and middle reaches of the Yangtze River (Fig. 3). After three consecutive nights’ flight, only 68.22% (292/406) valid endpoints located to within the suitable habitat, while 72.97% (270/370) did so after four consecutive nights. This may have been because the number of invalid endpoints increased as the number of nights increased (Fig. 3 and Table 3). However, the proportional difference after three and four nights was only 4.75%. Trajectories were calculated with different initial heights (See method, Table 3). Three consecutive nights’ flight was enough for most moths to finish their migration and find out their suitable habitat at all heights except at 1500 m (Table 3).

## Discussion

The morphological and physiological traits differences between migrant and non-migrant individuals of the same species have been studied widely^27^, especially within the context of the oogenesis-flight syndrome^22^. All these migratory traits were thought to be the consequence of physiology and morphology to adapt to migratory behavior^27^. Many studies on bird migration have shown that migration distance plays an important role in the expression of migratory traits, including fuel load and aerodynamic properties^28^,^29^,^30^,^31^. But studies on the driving forces or factors to shape insect migratory traits have been limited. In this study, most *C. medinalis* moths arrived at suitable breeding areas within three nights of simulated flight, and this migration duration agreed with our supposition as derived from previous studies on its physiological traits of ovarian development^25^. The distribution of suitable habitat presents the migration distance of flyers should cover, thus our results suggest for the first time, that migration distance represents an important role in determining the migration duration of *C. medinalis*, which may in turn shape its oogenesis-flight syndrome.

The distributions of rice host, temperature and relative humidity suggested that the expected destination of migratory *C. medinalis* moths was located in the lower and middle reaches of the Yangtze River during early and middle June. This result was in agreement with previous studies on migration processes in 1977, 1979 and 1980^14^,^15^. In these three years, *C. medinalis* moth population peaks occurred simultaneously in a wide area in this region during late May and the middle of June^14^,^15^. Based on these observations, its northward migration to eastern Asia was divided into 5 steps. Among these 5 steps, moths migrated from South China to the Yangtze River Valley in the third and fourth steps during late May to middle July^14^,^15^. The Yangtze River Valley is one of the most important rice-producing regions in eastern Asia covering the Jiangxi, Anhui, Jiangsu, Zhejiang and Shanghai Provinces^32^. Thus, it is a very important region available for *C. medinalis* to build and develop its population. Most individuals from the Yangtze River Valley population migrate to South China in autumn, except for a few individuals that migrate further north^33^. Compared with the other migration steps, the migration distance of the third step also was the longest. In this paper, only the third migration step during late May and middle June was studied. Our conclusions are very credible and meaningful because this third migration step is the most important for *C. medinalis* to exploit suitable habitat.

Due to changes in available resources through time and space, exploiting seasonal breeding habitats and escaping deteriorating conditions is the primary driver of the evolution of long-range migration^7^,^27^,^34^. Thus, the spatiotemporal distribution of suitable habitat has a great influence on the determination of migratory pathways, especially for monophagous or oligophagous insects. As expected, major rice crop pests such as *C. medinalis* and two rice planthopper species *Nilaparvata lugens* (Stål) and *Sogatella furcifera* (Horváth) share similar migration pathways in eastern Asia^14^,^15^,^35^,^36^. Previous studies have shown that population dynamics and migration patterns of *C. medinalis* and *N. lugens* have dramatically changed according to the change of rice planting systems^32^,^37^,^38^,^39^. In the Yangtze River valley, double-cropping rice was mainly planted from 1960 s to 1990 s, but double-cropping technique has now been greatly reduced and single-cropping practice has been rapidly increasing since 1997^32^. Due to the high suitability of single cropping rice, populations of *C. medinalis* and *N. lugens* grew so quickly that it caused serious local damage in these regions and mass emigration to the Yangtze River Delta^32^,^37^,^38^,^39^. It can therefore be inferred that migration distances were determined greatly by the distribution of suitable migratory habitat.

Previous studies also suggested that migration distance was related to body size, behavior strategy and morphological characteristics^40^,^41^. Compared to *C. medinalis*, rice planthoppers perform non-stop migration to finish their long-distance journey in one continuous flight. They use a different migration strategy to cover a similar migration distance. Study between silver Y moths and passerines showed that they achieved extremely similar levels of travel speed and orientation performance during their nocturnal migratory flights in the northern temperate zone by different behavioral strategies^3^,^4^. Therefore, it is unclear whether the migration distance was driven by a large variety in characteristics of morphology or animal behavior. Further work needs to be done to answer these complex questions. By contrast, our results suggest that migration distance could represent an important role to determine the migration duration of *C. medinalis* and to shape its oogenesis-flight syndrome. *C. medinalis* female moths developed their ovaries after three consecutive flight nights is a well-balanced surviving strategy to strike between migration and reproduction benefits. Hence, three nights’ flight seem to be an optimum solution of migration-reproduction trade-offs in energy allocation evolved in response to the natural selection on migration route and physiological traits.

## Method

### Survey Data of *C. medinalis*

Nine years of survey data of *C. medinalis* was collected from rice paddy fields in Nanning, Guangxi Autonomous Region in China (Fig. 1). Data was collected in May and June during the years 1978, 1979, 1980, 1981, 2004, 2005, 2007, 2010 and 2011, and comprises date recorded, moth abundance and ovary development. The abundance of moths was recorded based on a survey that was carried out in the morning, beginning at 7:00 AM (Beijing Time, BJT). A 2 m stick was swept across the canopy of rice plants, and the number of disturbed moths flying away were counted by eye. The survey covered a sweep area of at least 40 m^2^ of rice plants each morning. The number of moths per hectare was calculated and recorded. Ovary dissection data are available for the years 1978–1981 and 2007. Female moths of *C. medinalis* were collected from rice paddies and dissected daily to determine their stage of ovarian development according to the criteria described by Zhang *et al*.^26^. Females with ovarian development Level I–II were regarded as ‘sexually immature individuals’ and those with Level III–V were regarded as ‘sexually mature individuals’. The proportion of females with ovarian development categorised as Level–I, Level–II and ≥Level–III was tabulated.

### Meteorological data and modelling

The Weather Research and Forecasting (WRF) Model is a next-generation mesoscale numerical weather prediction system^42^. The model is designed to serve both atmospheric research and operational forecasting needs. The model was used to produce a high resolution atmospheric background for the trajectory analysis carried out in this study. The dimensions of the model domain were 99 × 84 grid points in a resolution of 30 km. Twenty-nine vertical layers were available and the model ceiling was 100 hPa. The scheme selection and parameters used for the WRF are listed in Supplementary Table S1. National Centers for Environmental Prediction (NCEP) Final Analysis (FNL) Data was used as the meteorological data for the model input. FNL is a six-hourly, global, 1-degree grid meteorological dataset. The model forecast time is 72 h with data outputs at 1 h intervals, for horizontal and vertical wind speeds, temperature and precipitation.

### Forward Trajectory Analysis

We estimated suitable migratory landing areas for *C. medinalis* moth by constructing their forward flight trajectories with the following assumptions. Firstly, no orientation pattern of *C. medinalis* was detected by radar, but moths displaced downwind^33^. Secondly, results from tethered flight experiments suggested that its self-powered flight speed was about 0.8 m/s and it cannot fly in an atmospheric temperature below 12.9 °C^43^. Thirdly, *C. medinalis* take off at dusk, about 20:00 BJT^33^,^43^,^44^. Fourthly, few *C. medinalis* moths have been observed by radar and aerial net after midnight^33^,^45^, and flight activity significantly decreases after 6 hours’ continuous flight (see Supplementary Fig. S1) leading most migrants to descend and land. Finally, the flight altitude of *C. medinalis* observed by radar was below 1000 m and most moths concentrated at about 500 m in autumn^33^,^45^. Forward trajectories on the first migration night began from Nanning with the start time taken at dusk (about 20:00 BJT), using five different initial flight altitude heights: 500, 750, 1000, 1250 and 1500 m above mean sea level. The air speed of migrants was wind speed plus with its self-powered flight (0.8 m/s). Trajectories were terminated when the air temperature fell below 12.9 °C or flight duration was up to six hours. On the second migration night, trajectory analysis continued from the last known locations of the first night’s trajectories with the same take off time and altitude. The complete forward trajectories analysis was calculated continuously for 5 consecutive nights.

This method was first introduced by Zhu and Liao^46^ with wind field and temperature data from the WRF output^46^,^47^. Further details for this method was described by Hu *et al*. & Lu *et al*.^12^,^47^. The script for calculating this trajectory was written in FORTRAN and run using the operating system Fedora 13 (Fedora Project, http://fedoraproject.org/).

### Expected destination of migratory *C. medinalis*

Previous studies have suggested that the optimal temperature and relative humidity ranges for rice leaf folder eggs were 22–31 °C and 77–100%, respectively^48^. In addition, *C. medinalis* moths prefer to lay eggs on rice plants at tillering stage and its larva mostly feed on rice plants between tillering and booting stages^49^,^50^. Adult moths emigrate when the rice host is at a deteriorative or heading stage^14^,^51^. Therefore, the expected destination of migratory moths should be to areas where rice plants were between tillering and booting stages. In South China, early and late season rice are planted successively in the same year, a planting system known as double-cropping rice^32^. The early rice must be harvested as early as possible in the summer, so that the fields can be ploughed and levelled for transplanting the late season rice. Rice plants are sensitive to low temperatures during the plant’s reproductive stage and the optimum temperature for indica rice heading is 25–35 °C^52^. Thus, no suitable rice hosts for *C. medinalis* larva would be available in areas where the average temperature was greater than 25 °C in South China in the first half of the year. Based on the above analyses, the expected destination of migratory *C. medinalis* is to areas where the average temperature is 22–25 °C and the relative humidity was greater than 77%. Daily long-term mean temperature and relative humidity were derived from the Japanese 55-year Reanalysis datasets (http://jra.kishou.go.jp/JRA-55). The meteorological information was applied to explore the range of suitable habitat of *C. medinalis* in South China. In addition, the rice planting schedules were surveyed at Nanning, Yongfu, Changsha, Huizhou and Huaining to check whether suitable rice host were growing (Fig. 1).

## Additional Information

**How to cite this article**: Wang, F.-Y. *et al*. Determining the migration duration of rice leaf folder (*Cnaphalocrocis medinalis* (Guenée)) moths using a trajectory analytical approach. *Sci. Rep.* **7**, 39853; doi: 10.1038/srep39853 (2017).

**Publisher's note:** Springer Nature remains neutral with regard to jurisdictional claims in published maps and institutional affiliations.

## Supplementary Material

Supplementary Tables and figures

## Figures and Tables

**Table 1:** Expected growth period of rice in selected sites in China during early and middle June.

Site	Latitude	Rice varieties	Growth period of rice	Suitable for *C. medinalis* feeding
Nanning	22.85	Early season rice	Heading	No
		Late season rice	Not yet planted	No
Yongfu	24.98	Early season rice	Booting	Yes
		Late season rice	Not yet planted	No
Wan’an	26.46	Early season rice	Booting to Heading	Yes
		Late season rice	Sowing	No
Changsha	28.20	Early season rice	Booting to Heading	Yes
		Middle season rice	Seedling	No
		Late season rice	Sowing	No
Huizhou	29.88	Middle season rice	Tillering	Yes
		Late season rice	Sowing	No
Anqing	30.51	Early season rice	Booting	Yes
		Middle season rice	Transplanting	No
		Late season rice	Sowing	No

**Table 2:** Abundance of *C. medinalis* moths and female ovarian development in Nanning, China.

Year	Period	No. of days	No. of moths observed per ha.	No. of moths dissected	Proportion (No.) of moths in each stage of ovarian development		
					Level - I	Level - II	≥Level - III
1978	1–16 Jun	15	616200	852	74.88 (638)	7.16 (61)	17.96 (153)
1979	5–19 Jun	8	64020	272	60.66 (165)	10.29 (28)	29.04 (79)
1980	30 May–16 Jun	9	138675	304	74.01 (225)	8.22 (25)	17.76 (54)
1981	13–21 Jun	9	298650	998	88.48 (883)	8.72 (87)	2.81 (28)
2007	27 May–6 Jun	7	374460	176	36.93 (65)	28.98 (51)	34.09 (60)

**Table 3:** Proportions of trajectory analysis endpoints that were located in the expected destinations of suitable habitat.

Height	Percent of valid endpoints (%)				
	1st night	2nd night	3rd night	4th night	5th night
500 m	10.20 (10/98)	30.99 (22/71)	41.18 (14/34)	26.67 (4/15)	0.00 (0/6)
750 m	15.89 (17/107)	46.81 (44/94)	67.09 (53/79)	59.62 (31/52)	56.41 (22/39)
1000 m	12.15 (13/107)	56.73 (59/104)	69.7 (69/99)	72.73 (64/88)	65.88 (56/85)
1250 m	7.48 (8/107)	57.41 (62/108)	73.83 (79/107)	78.1 (82/105)	75.00 (78/104)
1500 m	2.80 (3/107)	54.63 (59/108)	70.64 (77/109)	80.91 (89/110)	81.65 (89/109)
Total	9.70 (51/526)	50.72 (246/485)	68.22 (292/428)	72.97 (270/370)	71.43 (245/343)

Analysis was carried out on 5 consecutive nights and for five different altitude heights. This number is also expressed out of a total number of endpoints tested and given in parenthesis.
