# Supplementary material for: Determining the migration duration of rice leaf folder (Cnaphalocrocis medinalis (Guenée)) moths using a trajectory analytical approach
Source: Sci Rep. 2017 Jan 4;7:39853. doi: 10.1038/srep39853 (PMC5209671; doi:10.1038/srep39853)
Supplement: Supplementary Tables and figures [file srep39853-s1.doc]

**Determining the migration duration of rice leaf folder (*Cnaphalocrocis medinalis* (Guenée)) moths using a trajectory analytical approach**

Feng-Ying Wang^1,2, ♯^, Fan Yang^1,3, ♯^, Ming-Hong Lu^4^, Shan-Yu Luo^2^, Bao-Ping Zhai^1^, Ka-Sing Lim^5^, Caitríona E. McInerney^6^, Gao Hu^1, 5*^

*^1^ College of Plant Protection, Nanjing Agricultural University, Nanjing, China*

*^2^ Plant Protection Research Institute, Guangxi Academy of Agricultural Science and Technology, Nanning, China*

*^3^ Vegetable Research Institute, Wuhan Academy of Agricultural Science and Technology, Wuhan, China*

*^4^ Division of Pest Forecasting, China National Agro-Tech Extension and Service Center, Beijing, China*

*^5^Agro-Ecology Department, Rothamsted Research, Harpenden, Hertfordshire, UK*

*^6^Computational and Systems Biology, Rothamsted Research, Harpenden, Hertfordshire, UK*

♯ These authors contributed equally to this work

*Corresponding author: Gao Hu

Institution: Nanjing Agricultural University

Address: 1 Weigang Road, Nanjing 210095, China

Phone No.: +86(025)84395242

Fax No.: +86(025)84395242

E-mail address: hugao@njau.edu.cn

**Supplementary Table S1: Selection of scheme and parameters for the Weather Research and Forecasting (WRF) Model**

| Item | Domain 1 |
| --- | --- |
| Location | 25°N，108°E |
| The number of grid points | 99*84 |
| Distance ( km) between grid points | 30 |
| Layers | 29 |
| Map projection | Lambert |
| Microphysics scheme | WSM3 |
| Longwave radiation scheme | RRTM |
| Shortwave radiation scheme | Dudhia |
| Surface layer scheme | Monin-Obukhov |
| Land/water surface scheme | Noah |
| Planetary boundary layer scheme | YSU |
| Cumulus parameterization | Kain-Fritsch (new Eta) |
| Forecast time | 72 h |


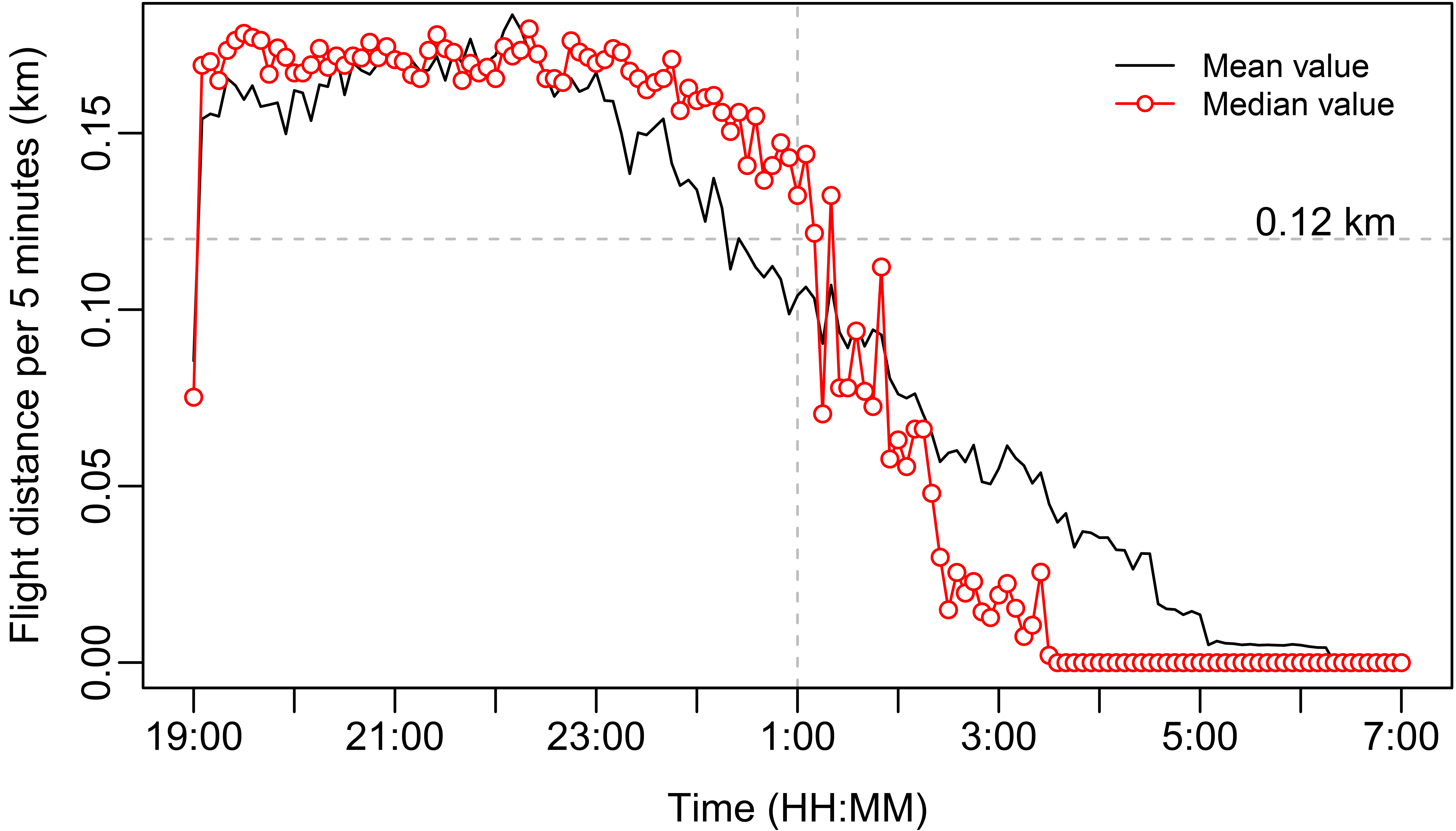


**Supplementary Fig. S1: Nocturnal flight activity of strong migratory *C. medinalis* moths.** A total of 56 healthy moths at two-days old were selected and tested. Flight tests were performed on a 24-channel computer-interfaced flight mill system (Jiaduo Co., Henan, China). A total of 34 of these moths were strong migrants with an accumulative flight duration greater than 130 minutes (Wang et al., 2010), including 20 females and 14 males. Flight activity is represented by flight distance in a 5-minutes period. The moth was identified at the active flight phase if it kept flying more than 2.5 minutes in each 5-minutes period, i.e. the flight distance was greater than 0.12 km with a flight speed of 0.8 m/s. The median values of their flight distance in each 5-minutes period showed that 50% moths can keep flying actively for 6.25 hours (Female: 5.75 hours, male: 6.25 hours).
